# Fear conditioning and extinction induce opposing changes in dendritic spine remodeling and somatic activity of layer 5 pyramidal neurons in the mouse motor cortex

**DOI:** 10.1038/s41598-019-40549-y

**Published:** 2019-03-15

**Authors:** Zhiwei Xu, Avital Adler, Hong Li, Luis M. Pérez-Cuesta, Baoling Lai, Wei Li, Wen-Biao Gan

**Affiliations:** 1 State Key Laboratory of Chemical Oncogenomics, Key Laboratory of Chemical Genomics, Peking University Shenzhen Graduate School, Shenzhen, 518055 China; 2 Skirball Institute, Department of Neuroscience and Physiology, Department of Anesthesiology, New York University School of Medicine, New York, NY 10016 USA

## Abstract

Multiple brain regions including the amygdala and prefrontal cortex are crucial for modulating fear conditioning and extinction. The primary motor cortex is known to participate in the planning, control, and execution of voluntary movements. Whether and how the primary motor cortex is involved in modulating freezing responses related to fear conditioning and extinction remains unclear. Here we show that inactivation of the mouse primary motor cortex impairs both the acquisition and extinction of freezing responses induced by auditory-cued fear conditioning. Fear conditioning significantly increases the elimination of dendritic spines on apical dendrites of layer 5 pyramidal neurons in the motor cortex. These eliminated spines are further apart from each other than expected from random distribution along dendrites. On the other hand, fear extinction causes the formation of new spines that are located near the site of spines eliminated previously after fear conditioning. We further show that fear conditioning decreases and fear extinction increases somatic activities of layer 5 pyramidal neurons in the motor cortex respectively. Taken together, these findings indicate fear conditioning and extinction induce opposing changes in synaptic connections and somatic activities of layer 5 pyramidal neurons in the primary motor cortex, a cortical region important for the acquisition and extinction of auditory-cued conditioned freezing responses.

## Introduction

Pavlovian fear conditioning has been widely used as an experimental paradigm for investigating mechanisms underlying fear memory formation and extinction^1^. In fear conditioning, a neutral stimulus (conditioned stimulus; CS) is paired with an aversive experience (unconditioned stimulus; US). After pairings, CS presented alone can trigger fear responses such as freezing. During fear extinction, repeated presentations of CS extinguish fear memory and decrease the freezing responses to CS^2^. A variety of pharmacological and electrophysiological studies have shown that the amygdala plays a critical role in the acquisition and expression of fear and extinction memories^3,4^. In addition to the amygdala, the hippocampus and multiple cortical regions are also involved in regulating conditioned fear and extinction responses^2,5–7^. For example, lesions and inactivation of the hippocampus disrupt the acquisition of contextual fear conditioning in rodents^8,9^, while inactivation of the anterior cingulate cortex (ACC) or auditory cortex impairs auditory-cued fear conditioning^10,11^. Furthermore, activation of the prelimbic prefrontal cortex causes sustained fear expression, while inactivation of the infralimbic prefrontal cortex impairs fear extinction^12,13^.

A typical fear response induced by fear conditioning is freezing behavior manifested as the absence of movement in response to conditioned stimuli^9^. The primary motor cortex is known to control both simple and complex motor behaviors^14–16^. Previous studies have suggested that fear conditioning increases *c-fos* expression in the rodent motor cortex^17,18^. In addition, the motor cortex receives axonal projections from various brain regions involved in fear conditioning and extinction including the amygdala, thalamus, prefrontal and auditory cortex^19–23^. Thus, the motor cortex is potentially involved in regulating the acquisition and extinction of conditioned freezing responses. However, the function of the primary motor cortex in fear conditioning and extinction remains to be investigated. Furthermore, previous studies have shown that fear conditioning causes elimination and formation of postsynaptic dendritic spines in the frontal association and auditory cortex respectively^24–28^. The remodeling of dendritic spines induced by fear conditioning is partially reversed after fear extinction^24,28^. Whether and how fear conditioning and extinction affect synaptic connections and neuronal activities in the primary motor cortex remain unknown.

In the present study, we show that inactivation of the primary motor cortex impairs auditory-cued fear conditioning and extinction. Similar to the frontal association cortex, fear conditioning induces the elimination of dendritic spines of layer 5 pyramidal neurons while fear extinction causes the formation of new spines near the site of previously eliminated spines in the primary motor cortex. We also show that fear conditioning reduces while fear extinction increases somatic activity in layer 5 pyramidal neurons in the motor cortex. Together, these findings suggest that changes in synaptic connections and neuronal activities in the motor cortex are important for regulating freezing responses after auditory-cued fear conditioning and extinction.

## Results

### Inactivation of the primary motor cortex impairs the acquisition and extinction of conditioned freezing responses

**Figure 1:**
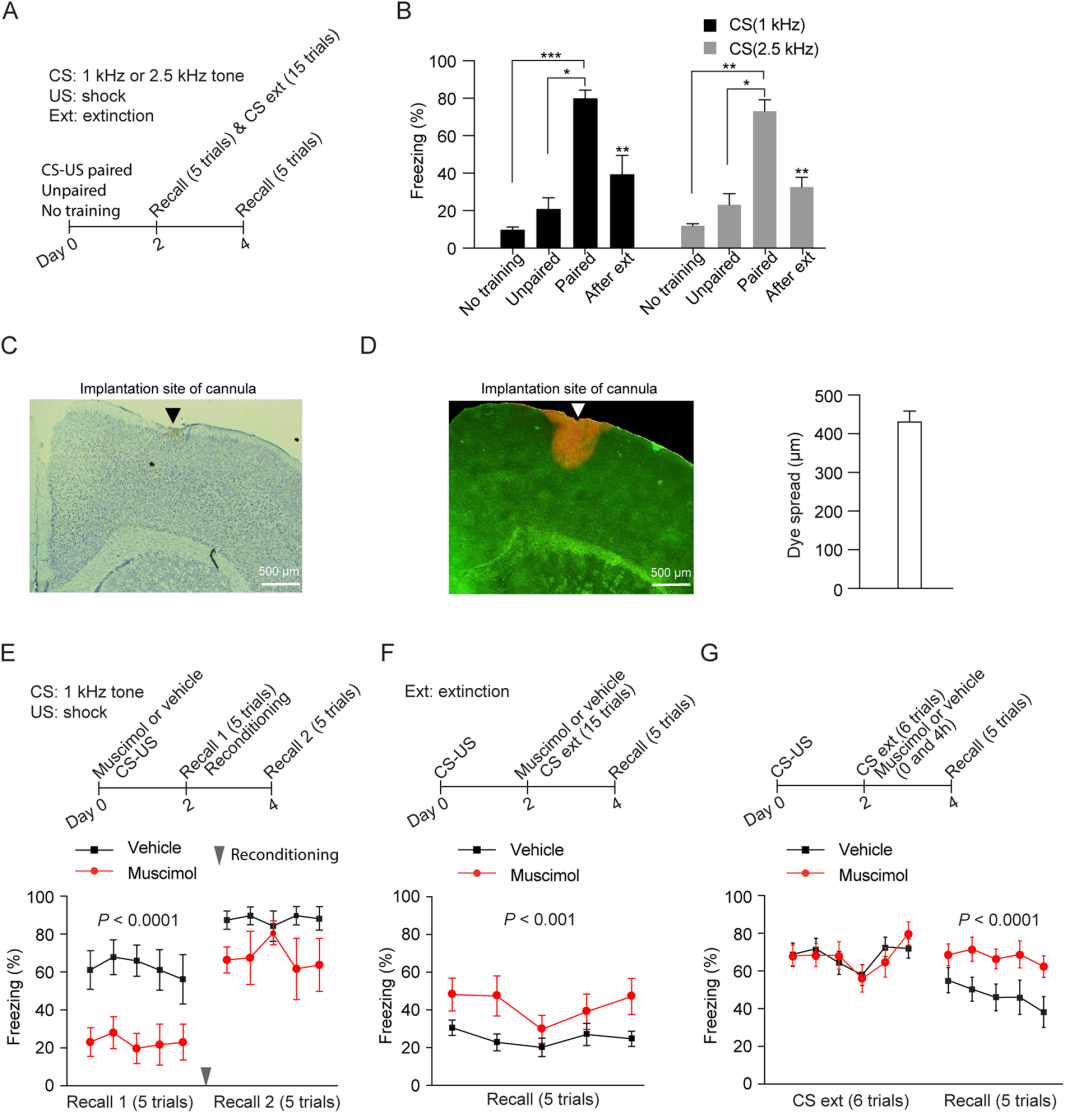
Inactivation of the primary motor cortex impairs conditioned freezing and extinction. (**A**) Schematic of experimental design. Mice were subjected to CS (1 or 2.5 kHz auditory tone) paired with US, unpaired stimuli or no training on day 0. These mice were subjected to recall test on day 2. The mice subjected to CS-US pairings were further subjected to fear extinction on day 2 and to recall test on day 4. (**B**) Mice subjected to CS-US pairings showed higher freezing response compared to mice subjected to unpaired stimuli and no training during the recall test on day 2. Fear extinction significantly decreased the conditioned freezing during the recall test on day 4 (n = 8 and 6 mice for CS (1 kHz)-US paired and extinction groups; n = 6 and 5 mice for CS (2.5 kHz)-US paired and extinction groups; n = 4–6 mice for unpaired and untrained groups). The average freezing response of 5 trials during the recall test was used in the comparison. (**C**) Nissl staining revealed the cannula position in the primary motor cortex. (**D**) Left: a representative brain section from YFP-expressing mice infused with Congo red through injection cannula inserted into the primary motor cortex. Right: the dye spread was determined by measuring mediolateral extent of Congo red in slice (n = 4 mice). (**E**) Top: schematic of experimental design. Muscimol (1 µl, 1 µg/µl) or vehicle was infused bilaterally into the primary motor cortex prior to fear conditioning by pairings CS (1 kHz auditory tone) and US on day 0. The same mice were subjected to recall test and reconditioning on day 2 and to recall test on day 4. Bottom: bilateral infusion of muscimol into the motor cortex before fear conditioning significantly reduced freezing during the recall test on day 2 (n = 10 and 7 mice for muscimol and vehicle groups respectively). After reconditioning, muscimol-infused mice showed higher freezing response during recall test on day 4 when compared to that on day 2 (n = 5 and 3 mice for muscimol and vehicle groups respectively during recall test on day 4). The freezing response of each trial during the recall test was used in the comparison. (**F**) Top: schematic of experimental design. Mice were subjected to CS-US pairings on day 0 and CS extinction with 15 tone presentations on day 2. Muscimol or vehicle was infused bilaterally into the primary motor cortex 1 hour before CS extinction on day 2. The same mice were subjected to recall test on day 4. Bottom: bilateral infusion of muscimol into the motor cortex before CS extinction significantly reduced freezing during the recall test on day 4 (n = 7 and 8 mice for muscimol and vehicle groups respectively). The freezing response of each trial during the recall test was used in the comparison. (**G**) Top: schematic of experimental design. Mice were subjected to CS-US pairings on day 0 and CS extinction with 6 tone presentations on day 2. Muscimol or vehicle was infused bilaterally into the primary motor cortex after extinction (twice, immediately and 4 hours) on day 2. The same mice were subjected to recall test on day 4. Bottom: after extinction, muscimol-infused mice showed significantly higher freezing during the recall test than vehicle-infused mice on day 4 (n = 9 and 10 mice for muscimol and vehicle groups respectively). Data are presented as mean ± S.E.M. *P < 0.05; **P < 0.01; ***P < 0.001.

Consistent with previous studies^29,30^, mice subjected to CS (1 or 2.5 kHz auditory tone) paired with US (footshock) showed significantly higher freezing during the recall test on day 2 than mice subjected to unpaired stimuli or no training (Fig. 1A,B, Kruskal-Wallis test: *H* = 13.35, *P* < 0.0001; multiple comparisons with two-stage linear step-up procedure of Benjamini, Krieger and Yekutieli: *P* < 0.001, paired vs. no training; *P* < 0.05, paired vs. unpaired; *P* = 0.3973, unpaired vs. no training; CS (1 kHz)) (Kruskal-Wallis test: *H* = 10.08, *P* = 0.0003; multiple comparisons with two-stage linear step-up procedure of Benjamini, Krieger and Yekutieli: *P* < 0.01, paired vs. no training; *P* < 0.05, paired vs. unpaired; *P* = 0.4985, unpaired vs. no training; CS (2.5 kHz)). To explore the involvement of the mouse primary motor cortex in fear conditioning, we inactivated the motor cortex through muscimol infusion (Methods and Fig. 1C,D) and subsequently examined freezing behavior after fear conditioning. When muscimol was infused into the primary motor cortex 1 hour before CS (1 kHz auditory tone)-US pairings on day 0, mice showed a significant decrease in freezing responses when compared to vehicle-infused mice during the recall test on day 2 (Fig. 1E, Muscimol: 1 µl, Two-Way ANOVA: drug: F_1,75_ = 41.06, *P* < 0.0001; trial: F_4,75_ = 0.2117, *P* = 0.9312; interaction: F_4,75_ = 0.1154, *P* = 0.9767) (Supplementary Fig. S1C, Muscimol: 0.2 µl, Two-Way ANOVA: drug: F_1,65_ = 6.931, *P* = 0.0106; trial: F_4,65_ = 1.245, *P* = 0.3009; interaction: F_4,65_ = 0.5115, *P* = 0.7274). Furthermore, 2 days after muscimol infusion, mice reconditioned with the same CS-US pairings showed higher freezing response during the recall test on day 4 when compared to that on day 2 (Fig. 1E, Two-Way ANOVA: reconditioning: F_1,50_ = 13.37; *P* = 0.0006; trial: F_4,50_ = 0.2915, *P* = 0.8822; interaction: F_4,50_ = 0.342; *P* = 0.8483). On the other hand, when muscimol was infused into the primary motor cortex immediately after fear conditioning on day 0, the freezing responses during the recall test on day 2 were comparable to that in vehicle-infused mice (Supplementary Fig. S2, Two-Way ANOVA: drug: F_1,65_ = 0.006, *P* = 0.9383; trial: F_4,65_ = 0.9201, *P* = 0.4577; interaction: F_4,65_ = 0.7673, *P* = 0.5504). In addition, when muscimol was infusedinto the primary motor cortex 1 hour before recall test, freezing responses during the recall test was similar to that in vehicle-infused mice (Supplementary Fig. S3, Two-Way ANOVA: drug: F_1,65_ = 1.644, *P* = 0.2044; trial: F_4,65_ = 0.7768, *P* = 0.5443; interaction: F_4,65_ = 0.5067, *P* = 0.7310). Taken together, these results suggest that the motor cortex is important for the acquisition of auditory-cued conditioned freezing response.

To investigate whether the motor cortex is also involved in fear extinction, mice were first fear conditioned with CS (1 or 2.5 kHz auditory tone)-US pairings and then subjected to fear extinction with repeated CS presentations. As expected, 2 days after fear extinction, mice showed significantly lower freezing response during the recall test on day 4 (Fig. 1A,B, Mann Whitney test, *P* < 0.01, CS (1 kHz); Mann Whitney test, *P* < 0.01, CS (2.5 kHz)). Importantly, when muscimol was infused into the primary motor cortex 1 hour before fear extinction, mice showed significantly higher freezing response during the recall test when compared to vehicle-infused mice (Fig. 1F, Two-Way ANOVA: drug: F_1,65_ = 15.1, *P* = 0.0002; trial: F_4,65_ = 1.176, *P* = 0.3296; interaction: F_4,65_ = 0.4239, *P* = 0.7909). Furthermore, infusion of muscimol into the primary motor cortex immediately and 4 hours after CS extinction also significantly increased freezing response during the recall test when compared to vehicle-infused mice (Fig. 1G, Two-Way ANOVA: drug: F_1,85_ = 21.48, *P* < 0.0001; trial: F_4,85_ = 0.8736, *P* = 0.4833; interaction: F_4,85_ = 0.178, *P* = 0.9492). Together, these results suggest that the primary motor cortex plays important roles in regulating the freezing responses after both fear conditioning and extinction.

### Fear conditioning and extinction induce spine elimination and formation of apical dendrites of layer 5 pyramidal neurons in the mouse primary motor cortex respectively

**Figure 2:**
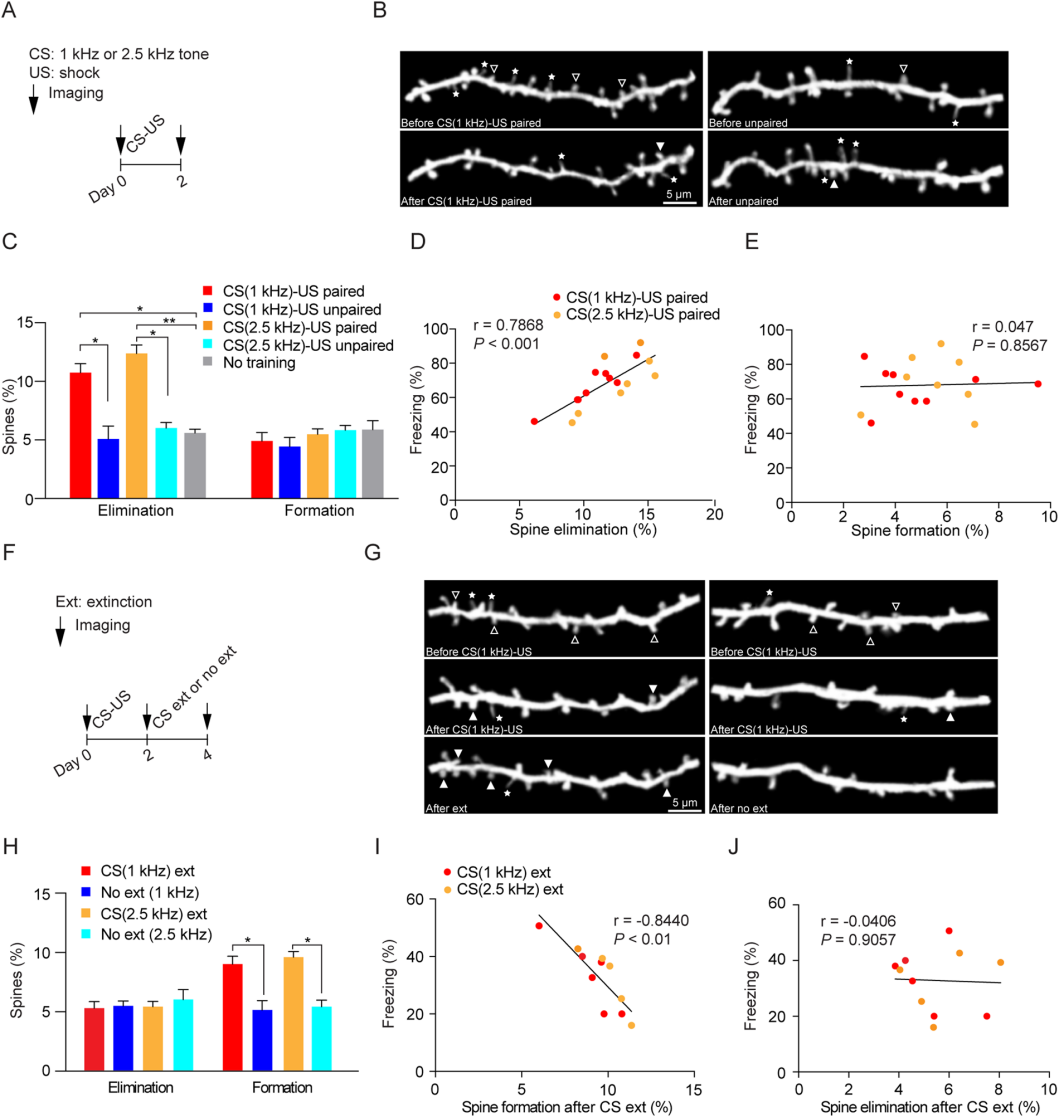
Fear conditioning and extinction increase spine elimination and formation of apical dendrites of layer 5 pyramidal neurons in the mouse primary motor cortex respectively. (**A**) Schematic of experimental design. YFP-expressing transgenic mice were subjected to CS (1 or 2.5 kHz tone) paired with US, unpaired stimuli or no training. Imaging was performed before and 2 days after fear conditioning. (**B**) Representative images of spine formation and spine elimination before and after CS-US pairings or before and after unpaired stimuli. Eliminated spines were identified in the initial view but not in the second view. Newly formed spines were identified in the second view but not in the initial imaging view. The hollow triangles and solid triangles indicate eliminated spines and newly formed spines respectively. The stars indicate filopodia. (**C**) The rate of dendritic spine elimination, but not formation, was significantly higher in mice subjected to CS-US pairings than that in mice subjected to unpaired stimuli or no training (n = 9 and 3 mice for CS (1 kHz)-US and unpaired groups; n = 9 and 4 mice for CS (2.5 kHz)-US paired and unpaired groups; n = 4 mice for untrained group). The rate of spine elimination or formation after fear conditioning was calculated as the number of eliminated spines or newly formed spines after fear conditioning divided by the number of pre-existing spines in the initial imaging view before fear conditioning. (**D-E**) Correlation of spine elimination (**D**) and spine formation (**E**) with freezing. The rate of spine elimination, but not formation, after fear conditioning was positively correlated with the freezing responses to CS. (**F**) Schematic of experimental design. Mice were first subjected to CS (1 or 2.5 kHz tone)-US pairings and then subjected to CS extinction or no extinction training. Imaging was performed after CS-US pairings and after CS extinction or no extinction to determine the spine remodeling after CS extinction. (**G**) Representative images of spine formation and elimination before and after CS-US pairings, and after CS extinction or no extinction. The hollow triangles and solid triangles indicate eliminated spines and newly formed spines respectively. The stars indicate filopodia. (**H**) The rate of spine formation, but not elimination, was significantly higher in the extinction group as compared to the no extinction group (n = 6 and 5 mice for CS (1 kHz) extinction and no extinction groups; n = 5 and 4 mice for CS (2.5 kHz) extinction and no extinction groups). The rate of spine elimination or formation after fear extinction was calculated as the number of eliminated spines or newly formed spines after fear extinction divided by the number of pre-existing spines in the imaging view before fear extinction. (**I**,**J**) The rate of spine formation (**I**) but not elimination (**J**) was inversely correlated with the freezing response to CS after extinction. Data are presented as mean ± S.E.M. *P < 0.05; **P < 0.01.

Previous studies have shown that fear conditioning results in dendritic spine elimination and formation on apical dendrites of layer 5 pyramidal neurons in the mouse frontal association and auditory cortex, respectively^24–28^. To investigate the impact of fear conditioning on synaptic connections in the primary motor cortex, we examined postsynaptic dendritic spines of layer 5 pyramidal neurons before and after fear conditioning with *in vivo* two-photon microscopy^24,31^. We found that the rate of spine elimination, but not formation, over 2 days was significantly higher in mice subjected to CS (1 or 2.5 kHz auditory tone) paired with US than in control mice subjected to unpaired stimuli or no training (Fig. 2A–C, Kruskal-Wallis test: *H* = 9.839, *P* = 0.001; multiple comparisons with two-stage linear step-up procedure of Benjamini, Krieger and Yekutieli: *P* < 0.05, paired vs. no training; *P* < 0.05, paired vs. unpaired; *P* = 0.7658, unpaired vs. no training; CS (1 kHz)) (Kruskal-Wallis test: *H* = 12.91, *P* < 0.0001; multiple comparisons with two-stage linear step-up procedure of Benjamini, Krieger and Yekutieli: *P* < 0.01, paired vs. no training; *P* < 0.05, paired vs. unpaired; *P* = 0.5962, unpaired vs. no training; CS (2.5 kHz)). Furthermore, the rate of spine elimination, but not formation, after fear conditioning was significantly correlated with freezing responses to CS (Fig. 2D, Pearson correlation: r = 0.7868, *P* < 0.001; Fig. 2E, Pearson correlation: r = 0.047, *P* = 0.8567). Consistently, we found that mice injected with muscimol showed not only reduced freezing response (Fig. 1E), but also reduced spine elimination 2 days after fear conditioning when compared to vehicle-injected mice (Supplementary Fig. 4, Mann Whitney test, *P* < 0.05).

Previous studies have shown that fear extinction induces dendritic spine formation and elimination of layer 5 pyramidal neurons in the frontal association cortex and auditory cortex respectively^24,28^. To investigate how fear extinction changes spine remodeling in the motor cortex, mice were first fear conditioned by CS-US pairings and then subjected to fear extinction 2 days later (Fig. 2F). We found that spine formation, but not elimination, in the primary motor cortex was significantly higher in the extinction group (CS 1 or 2.5 kHz auditory tone) as compared to that in the no extinction group (Fig. 2G,H, Mann Whitney test, *P* < 0.05, CS (1 kHz); Mann Whitney test, *P* < 0.05, CS (2.5 kHz)). Furthermore, the rate of spine formation, but not elimination, was inversely correlated with freezing response to CS after fear extinction (Fig. 2I, Pearson correlation: r = −0.844, *P* < 0.01; Fig. 2J, Pearson correlation: r = −0.0406, *P* = 0.9057). Thus, similar to the frontal association cortex, fear conditioning and extinction cause spine elimination and formation of layer 5 pyramidal neurons in the primary motor cortex.

### Spines eliminated after fear conditioning are apart from each other and newly formed spines after fear extinction are located near the site of previously eliminated spines

**Figure 3:**
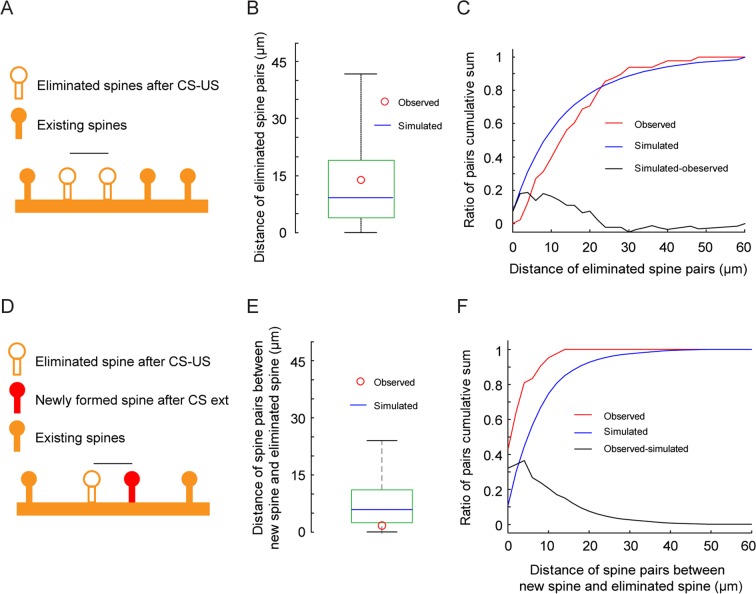
Spines eliminated after fear conditioning are apart from each other along dendrites while newly formed spines after fear extinction are located near the site of previously eliminated spines. (**A**) Schematic of measurement of the distance between eliminated spines after CS-US pairings. The yellow solid circles represent existing spines. The yellow hollow circles represent eliminated spines. The black line represents the measured distance between eliminated spines. (**B**) Box plot showing the distribution of distance of spine pairs between eliminated spines in the simulation. The green square shows the 25–75th percentile of the distance, the black line shows the 99th percentile and the blue line shows the median. The red circle shows the median of the observed eliminated spine pair distances. The median of the observed data (n = 62) was larger than the median of the simulated data. (**C**) Cumulative distribution of the distance between eliminated spine pairs after CS-US in the data (observed, red) and between simulated spine pairs (simulation mean over 1000 shuffles, blue line). (**D**) Schematic of measurement of the distance of spine pairs between eliminated spines after CS-US and new spines after extinction. The yellow solid circles represent existing spines. The yellow hollow circle represents the eliminated spine. The red solid circle represents the newly formed spine. The black line represents the measured distance of spine pairs between the eliminated spine after CS-US and the new spine after extinction. (**E**) Box plot showing the distribution of the distance of spine pairs between eliminated spines after CS-US and simulated new spines. The green square shows the 25–75th percentile of the distance, the black line shows the 99th percentile and the blue line shows the median. The red circle shows the median of the observed newly formed-eliminated spine pair distances. The median of the observed data (n = 42) was shorter than the median of the simulated data. (**F**) Cumulative distribution of the distance of spine pairs between eliminated spines after CS-US and newly formed spins after extinction (observed, red) and between eliminated spines after CS-US and simulated new spines (simulation, blue).

It has been suggested that clustered synaptic plasticity on individual dendrites may facilitate memory storage and/or recall^32–35^. To investigate whether the eliminated spines induced by fear conditioning were clustered or not, we examined the distance between eliminated spines on dendritic segments in response to fear conditioning and compared them to random simulation data. We found that the distance between eliminated spine pairs was significantly larger than that between randomly simulated eliminated spine pairs along dendrites (Fig. 3A,B, Mann Whitney test, *P* < 0.05). Moreover, cumulative sum of the distance showed that the probability density of the observed and simulated data was different (Fig. 3C, Kolmogorov-Smirnov test, *P* < 0.05). These results indicate that eliminated spines tend to be apart from each other along dendrites rather than randomly distributed.

Previous research in the frontal association cortex has shown that a large fraction of new spines induced by fear extinction are formed near the site where spines are eliminated by fear conditioning^24^. Consistent with the study, we found the distance between newly formed spines after extinction and eliminated spines after CS-US pairings was significantly shorter than that between simulated new spines and eliminated spines after CS-US pairings (Fig. 3E, Mann Whitney test, *P* = 2.1371e-14). Moreover, cumulative sum of the distance showed that the probability density of the observed and simulation data was different (Fig. 3F, Kolmogorov-Smirnov test, *P* = 8.8067e-12). Thus, similar to the frontal association cortex, new spines induced by CS extinction in the primary motor cortex tend to grow near spines eliminated after fear conditioning.

### Fear conditioning and extinction decrease and increase activities of layer 5 pyramidal neurons in the primary motor cortex respectively

**Figure 4:**
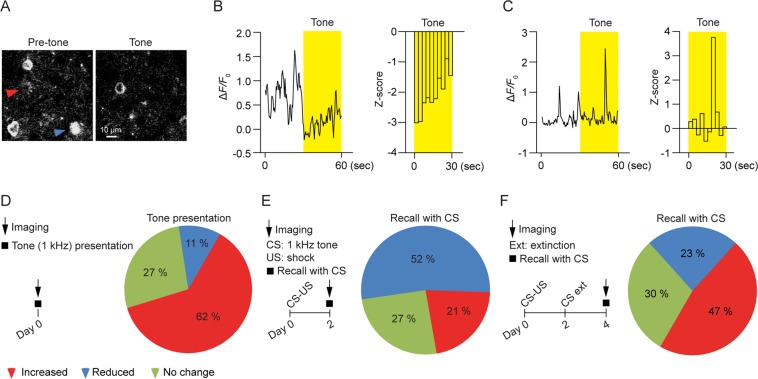
Fear conditioning and extinction decrease and increase somatic activities of layer 5 pyramidal neurons in the motor cortex respectively. (**A**) Representative images of calcium activities of layer 5 pyramidal neurons in GCaMP-expressing transgenic mice. The blue and red triangles indicate somas showing reduced and increased activities to tone respectively as compared to the pre-tone period. (**B**) The response of the labeled soma in (A, blue) with reduced activity to tone. Changes in the somatic calcium level were measured by Δ*F*/*F*_0_ (left) and z-score transformation (right). Yellow bars represent tone presentation. (**C**) The response of the labeled soma in (A, red) with increased activity to tone. Changes in somatic calcium level were measured by Δ*F*/*F*_0_ (left) and z-score transformation (right). Yellow bars represent tone presentation. (**D**) Left panel: schematic of experimental design. Somatic calcium activities of layer 5 pyramidal neurons were imaged with or without tone presentation. Right panel: the majority of layer 5 pyramidal neurons exhibited increased somatic calcium activities in response to tone compared to the period of pre-tone (n = 84 somas from 3 mice). (**E**) Left panel: schematic of experimental design. 2 days after CS-US pairings, somatic calcium activities of layer 5 pyramidal neurons were imaged with or without tone presentation. Right panel: the majority of layer 5 pyramidal neurons exhibited reduced somatic calcium activities in response to CS when compared to the period of pre-CS (n = 85 somas from 4 mice). (**F**) Left panel: schematic of experimental design. Mice were fear conditioned by CS-US pairings and then subjected to CS extinction. 2 days after CS extinction, somatic calcium activities of layer 5 pyramidal neurons were imaged with or without tone presentation. Right panel: a small percentage of layer 5 pyramidal neurons exhibited reduced somatic calcium activities in response to CS when compared to the period of pre-CS (n = 60 somas from 4 mice).

To investigate the impact of fear conditioning and extinction on the motor cortex further, we performed calcium imaging to examine the activity of GCaMP6-expressing layer 5 pyramidal neurons in the primary motor cortex. In this experiment, mice were head-restrained and subjected to tone presentations under a two-photon microscope. Layer 5 pyramidal neuronal activity was measured by somatic calcium imaging before and during tone presentation over a 60-second period (Fig. 4A–C). We found that the majority of layer 5 pyramidal neurons showed an increase in the somatic calcium activity in response to auditory tone (1 kHz) when compared to that during the period of pre-tone (Fig. 4D; increased activity: ~62%; reduced activity: ~11%; n = 84). When mice were first subjected to CS-US pairings and calcium imaging was performed 2 days later, larger percentage of layer 5 pyramidal neurons in the motor cortex showed a reduction in the somatic calcium activity in response to CS relative to pre-CS (Fig. 4E; reduced activity: ~52%, chi-square test, χ^2^ = 33.07, df = 1, *P* < 0.0001, compared to that before fear conditioning; increased activity: ~21%, χ^2^ = 28.88, df = 1, *P* < 0.0001; n = 85). On the other hand, when fear-conditioned mice were subjected to fear extinction and calcium imaging was performed 2 days later, a smaller percentage of neurons exhibited reduced activities in response to CS relative to pre-CS (Fig. 4F; reduced activity: ~23%, χ^2^ = 11.85, df = 1, *P* < 0.001, compared to that after fear conditioning; increased activity: ~47%, χ^2^ = 10.55, df = 1, *P* < 0.01; n = 60). Together, these results indicate that fear conditioning and extinction decrease and increase the activities of layer 5 pyramidal neurons in the primary motor cortex respectively.

## Discussion

In this study, we show that muscimol infusion into the mouse primary motor cortex significantly impairs both the acquisition and extinction of auditory cue-conditioned freezing responses. Similar to the frontal association cortex, fear conditioning causes spine elimination while fear extinction causes spine formation of layer 5 pyramidal neurons in the motor cortex. Concomitantly, fear conditioning and extinction decrease and increase the activity of these neurons respectively. Together, these findings indicate that the primary motor cortex undergoes opposing changes in synaptic remodeling and neuronal activity after fear conditioning and extinction and is important for the acquisition and extinction of conditioned fear responses.

The primary motor cortex is a crucial area involved in the execution of voluntary movements^14–16,36–38^. Direct electrical stimulation of the motor cortex can cause forelimb and whisker movements^39,40^. Electrophysiological recordings suggest that neuronal activity in the primary motor cortex encodes limb movements and movement sequences^36,37^. Consistently, our results with the inactivation of the primary motor cortex suggest important roles of this cortical region in the acquisition and extinction of conditioned freezing responses. We show that fear conditioning decreases activities of layer 5 pyramidal neurons in response to CS while fear extinction increases activities of these neurons in the motor cortex. Because layer 5 pyramidal neurons are the major output neurons in the motor cortex with projections to subcortical areas including the spinal cord, basal ganglia and thalamus^41–43^, it is possible that decreased activities of layer 5 pyramidal neurons after fear conditioning may reduce neuronal activity in subcortical areas and modulate conditioned freezing responses. On the other hand, increased activities of layer 5 pyramidal neurons after fear extinction may facilitate the movement of mice in response to CS.

Recent studies have shown that fear conditioning induces spine elimination or formation in the frontal association cortex and auditory cortex respectively^24–28^. Similar to the frontal association cortex (but different from the auditory cortex), our results show that fear conditioning induces spine elimination of layer 5 pyramidal neurons in the primary motor cortex and suggest that fear-related information is likely stored in multiple brain regions. The relationship between synaptic/neuronal changes in various brain regions remains unclear. Because motor cortex receives axonal projections from the amygdala, thalamus, prefrontal cortex and auditory cortex^19–23^, it is possible that these brain regions converge to cause plastic changes in the motor cortex, which in turn regulate freezing responses. It would be interesting to investigate whether fear conditioning eliminates synaptic connections from these brain regions, thus decreasing the activities of layer 5 pyramidal neurons and increasing the freezing response to CS. It would also be interesting to examine whether fear extinction may re-establish these connections to increase the activity of layer 5 pyramidal neurons. Future studies are needed to investigate the origin of axonal inputs to dendritic spines eliminated and formed to better understand how fear-related information is stored in the brain.

Many lines of evidence suggest that synaptic plasticity may occur in a clustered fashion during development and learning^32–35^. It has been shown that highly clustered synapse formation occurs between the CA3-CA1 hippocampal neurons with the same developmental history^44^. In the motor cortex, new spines are formed after motor learning in adjacent positions along the dendrites^45^. Furthermore, *in vitro* and *in vivo* studies have shown that synapses tend to be activated in clusters in slice cultures of developing hippocampus and in the barrel cortex^46,47^. In contrast to synaptic clustering under these conditions, we found eliminated spines induced by fear conditioning are further apart rather than being clustered or randomly distributed. Previous studies have shown that LTP induction causes the diffusion of calcium-dependent RAS and RhoA within the activated spine into adjacent spines^48,49^. It has been reported that LTD induces input-specific spine shrinkage at individual stimulated spines but not unstimulated spines^50^. Future studies of signaling mechanisms at the level of individual dendrites are needed to understand the dispersed spine elimination after fear conditioning. It is important to mention that our studies are limited to dendritic spine plasticity on the apical dendrites of layer 5 pyramidal neurons in the motor cortex. It remains to be investigated whether and how fear learning modifies synaptic changes on basal dendrites of layer 5 pyramidal neurons and on dendrites of other cell types in the motor and other cortical regions. The answers to these questions would help to better understand how experience-dependent synaptic changes contribute to fear memory consolidation.

It is generally believed that extinction represents new learning that inhibit original fear memory^2,51^. Behavioral studies have provided strong evidence that conditioned fear response can recover after extinction with passage of time and contextual shift^52,53^. In contrast to new learning mechanisms of extinction, recent studies have also suggested that extinction can reverse neuronal changes induced by fear conditioning under certain conditions^7,24,28,54–57^. It has been shown that fear conditioning and extinction increase and decrease neuronal activity in the amygdala respectively^56^. In the frontal association cortex, fear conditioning increases dendritic spine elimination while fear extinction induces new spine formation of layer 5 pyramidal neurons in a location- and cue-specific manner^24^. Furthermore, in the auditory cortex, fear conditioning induces new spine formation while extinction causes preferential elimination of the new spines^28^. Similar to the frontal association cortex, our results in the motor cortex indicate that fear conditioning and extinction cause dendritic spine elimination and formation respectively. Furthermore, new spines after fear extinction are close to the site of eliminated spines after fear conditioning. Together, our results in the primary motor cortex suggest that fear extinction may partially reverse fear conditioning-induced changes at the level of synapses in multiple brain regions.

## Materials and Methods

### Animals

Mice expressing YFP (H line) in layer 5 pyramidal neurons were obtained from the Jackson Laboratory, and mice expressing GCaMP6 in layer 5 pyramidal neurons were generated in the transgenic facility at NYU medical center. Mice were group-housed in the animal facility with all experiments approved by both NYU School of Medicine’s and Peking University’s Institutional Animal Care and Use Committees (IACUC). All experiments were performed in accordance with the Institutional guidelines.

### Behavior

Fear conditioning and extinction were performed in a training cage within a sound-attenuating box (Coulbourn Instruments). Behavior was recorded by low-light video cameras. Stimulus (auditory cue or shock) presentation was automated by using Actimetrics FreezeFrame software (Coulbourn Instruments). The cage was cleaned with water before experiments.

Fear conditioning was performed in a training cage equipped with stainless-steel shocking grids connected to a precision feedback current-regulated shocker. Mice were habituated for 2 min on shocking grids and then received seven pairings of a 30-s, 1 or 2.5 kHz auditory cue (80 dB tone, cage enclosed) co-terminating with a 2-s, 0.5-mA footshock. The intertrial interval was 90 s. One minute after training, mice were returned to their home cages. For the unpaired group, mice received seven presentations of auditory tone and shock in unpaired manner. The tones and shocks were separated by random intervals of 20–40 s. For the untrained group, mice were habituated in the training cage without tone or shock presentations.

Recall test was performed in a cage with a different context (scent:1% Pinesol) from the training cage used in fear conditioning. The cage was equipped with non-shocking grids. Mice were habituated for 2 min and received five presentations of the same auditory tone used in fear conditioning. The inter-trial interval was 90 s. One minute after test, mice were returned to their home cages.

Fear extinction was performed in a cage with a different context from the training cage used in fear conditioning. The cage was equipped with non-shocking grids. Mice were habituated for 2 min and received fifteen presentations of the same auditory tone used in fear conditioning. The inter-trial interval was 90 s. One minute after training, mice were returned to their home cages.

Recall of fear extinction was performed in a cage different from the cage used in fear extinction. Mice were habituated for 2 min and received five presentations of the same auditory tone. The inter-trial interval was 90 s. One minute after recall, mice were returned to their home cages.

Reconditioning was performed in a cage with a different context (scent: ethanol) from the training cage used in fear conditioning. The cage was equipped with shocking grids. Mice were habituated for 2 min on shocking grids and then received seven pairings of the auditory cue co-terminating with footshock as trained in fear conditioning. The intertrial interval was 90 s. One minute after reconditioning, mice were returned to their home cages.

### Inactivation of the primary motor cortex

1-month-old C57BL/J mice were anaesthetized with ketamine and xylazine and a guide cannula was implanted bilaterally into the primary motor cortex (+1.2 mm from Bregma, +1.6 mm from the midline, ~500 µm in depth). The guide cannula was held with dental acrylic cement. The mice were subjected to fear conditioning training three days after surgery. To examine the role of the primary motor cortex in fear conditioning, muscimol (1 µl or 0.2 µl, 1 µg/µl) was infused into the motor cortex bilaterally within 2–5 min, 1 hour prior to fear conditioning on day 0 or immediately after fear conditioning. For microinfusion, an injection cannula of the same length as the guide cannula was inserted into the guide cannula. Muscimol infusion was performed through the injection cannula connected with a microsyringe driven by a microinfusion pump. After fear conditioning, mice were subjected to recall test and reconditioning on day 2 and recall test again on day 4. To examine the role of the primary motor cortex in the acquisition of fear extinction, mice were subjected to fear conditioning on day 0 and subjected to fear extinction with 15 tone-alone presentations on day 2. Muscimol was infused into the motor cortex 1 hour prior to fear extinction. To examine the role of the primary motor cortex in the consolidation of fear extinction, muscimol was infused immediately and 4 hours after fear extinction with 6 tone-alone presentations on day 2. 6 tone presentations and two injections of muscimol were chosen in order to change the activity of the motor cortex during the time window of memory consolidation^30,58,59^. Mice were then subjected to recall test on day 4.

To determine the position of cannula, mice were sacrificed and brains were cut into sections at 30 µm. Sections were mounted on slides and stained with tolridine blue. Cannula position was identified using a light microscope. In the sections showing the position of cannula insertion, the primary motor cortex was identified as the area 1–2.7 mm lateral to the longitudinal fissure and ~1 mm from the bregma with the aid of atlas of the mouse brain^60^.

To determine the spread of muscimol, 1 µl Congo red (0.5%) was infused within 5 min through injection cannula inserted into the primary motor cortex (+1.2 mm from Bregma, +1.6 mm from the midline, ~500 µm in depth) of YFP-expressing mice. 1 hour after the infusion, mice were sacrificed and brains were cut into sections at 50 µm. Sections were then imaged using a fluorescence microscope. The dye spread was estimated by measuring the mediolateral extent of Congo red in slices.

### Imaging and analysis of spine remodeling

Details of the procedures for *in vivo* imaging and data analysis have been described in the previous studies^24,31^. Briefly, 1-month-old YFP-expressing mice were anaesthetized with ketamine and xylazine. Skull was glued to a stainless steel plate and a small region of the primary motor cortex (~200 µm in diameter; +1.2 mm from Bregma, +1.2 mm from the midline) was thinned to about 20 µm using a high-speed microdrill. The apical dendrites of layer 5 pyramidal neurons were imaged with a two-photon microscope with the laser wavelength tuned to 920 nm. The map of the brain vasculature was used to relocate the imaged region. The region of interest was re-thinned with microsurgical blades for repeated imaging.

Data analysis was performed with the ImageJ software as described before^24,31^. Briefly, the same dendritic segments were identified from three-dimensional stacks taken from all imaging views. Three-dimensional stacks were used to ensure that tissue movements and rotation between imaging intervals did not influence spine identification. The number and location of dendritic spines were identified in each view. Filopodia were identified as long thin structures with head diameter to neck diameter <1.2 and length to neck diameter >3. The remaining dendritic protrusions were classified as spines. Spines were considered the same between two imaging views if they were within 0.7 µm of their expected positions.

On average, ~150 spines and 6 dendrites were analyzed from each animal to calculate spine formation and elimination. The rate of spine formation or elimination was calculated as the number of newly formed spines or eliminated spines divided by the number of pre-existing spines. Newly formed spines were identified to appear in the second view but not in the initial imaging view. Eliminated spines were identified to appear in the initial view but not in the second view.

To examine the effects of muscimol on fear conditioning-induced spine elimination, spine imaging was performed in awake, head-restrained mice. 24 hours before imaging, mice were anesthetized and a head holder was attached as described before^31^. Specifically, the mouse head was shaved and the skull surface was exposed. A head holder composed of two parallel micro-metal bars was attached to the animal’s skull to help restrain the animal’s head and reduce motion-induced artifact during imaging^31^. A small region of the primary motor cortex (~200 µm in diameter; +1.2 mm Bregma, +1.2 mm from the midline) was thinned to about 20 µm using a high-speed microdrill. For muscimol administration, a glass microelectrode was bilaterally inserted through a bone flap (~50 µm in diameter) into the cortex (~500 µm in depth) with an angle of 45° towards and ~100 µm away from the imaging area^61^. One hour before fear conditioning, muscimol (1 µl, 1 µg/µl) was injected via pressure injections through the glass microelectrode into the motor cortex of head-fixed mice.

### Simulation

We simulated the locations of spines (eliminated or newly formed) along dendritic segments and compared the distances between the simulated spines with the distances observed in the data. All distance measurements in both the data and the simulation were done under the approximation of dendritic shafts as one dimensional. Therefore, the distance between two spines emerging from the same location on the dendritic shaft but pointing to different locations was determined to be zero. The simulation was conducted under the assumption that spines are eliminated or formed independently and randomly along dendritic segments. To simulate the distances between eliminated spine pairs, we randomly and independently generated spine locations on dendritic segments according to the dendrites’ lengths and the number of eliminated spines from each dendrite as measured in the data. Next, we calculated the closeted distance between each eliminated spine pair in the observed data and in the simulated data. Finally, we repeated this process for the simulated data 1000 times to produce the null distribution of eliminated spine distances along dendritic segments. We measured the medians of eliminated spine distances and compared the medians of the simulated data with the observed data using Mann-Whitney U-test. We compared the cumulative probabilities of these two distributions using Kolmogorov-Smirnov test. To simulate the distances between eliminated and newly formed spine pairs we performed a similar analysis as described above, only we simulated the locations of newly formed spines on the dendrites and calculated their distance to the locations of the observed eliminated spines in the data. Analysis was done using custom-written Matlab Codes.

### Imaging and analysis of somatic calcium activity

1-month-old GCaMP6-expressing transgenic mice were used for calcium imaging experiments. 24 hours before imaging, mice were anesthetized and a head holder was attached. A craniotomy was made above the primary motor cortex. A glass coverslip was placed on the craniotomy and was glued to the skull to reduce the brain motion as described before^31^.

Calcium imaging was performed in awake, head-restrained mice. To examine calcium activity of somas in response to tone, mice were exposed to 2–3 tone presentations (80 dB, 30 s). The somas of layer 5 neurons were imaged under a two-photon microscope with the laser wavelength tuned to 920 nm. The imaging period was divided into a 30 s period of pre-tone and a 30 s period of tone presentation. The inter-trial interval was 120 s.

To examine calcium activities after fear conditioning, mice were subjected to fear conditioning and somatic calcium activities of layer 5 pyramidal neurons were imaged with or without tone presentations 2 days after fear conditioning. To examine calcium activities after fear extinction, mice were subjected to fear conditioning and then to fear extinction. Somatic calcium activities of layer 5 pyramidal neurons were imaged with or without tone presentations 2 days after fear extinction.

Data analysis was performed using ImageJ software. The fluorescence time course of each labeled cell was measured by averaging all pixels within the circular ROIs covering the soma. The Δ*F*/*F*_0_ was calculated as (*F*-*F*_0_)/*F*_0_, where *F*_0_ is determined by averaging the baseline fluorescence signal between peak fluorescence signal over a 1 min imaging period. To examine changes in soma activity, 30 s tone presentation period was divided into ten bins equally. A z-score for each bin was calculated relative to ten bins of pre-tone period. Neurons were classified as showing increased responses if any tone bin exceeded 1.6. Neurons were classified as showing reduced responses if any tone bin exceeded −1.6.

### Statistics

All data were presented as the mean ± S.E.M. Tests for differences between groups were performed using non-parametric tests or ANOVA. Chi-Square test was used to compare the percentage of neurons under different conditions. Significant levels were set at *P* < 0.05. All statistical analyses were performed using GraphPad Prism.

## Supplementary information

Supplementary figures

## Data Availability

The data that support the findings of this study are available from the corresponding author upon request.
