# Supplementary material for: Fear conditioning and extinction induce opposing changes in dendritic spine remodeling and somatic activity of layer 5 pyramidal neurons in the mouse motor cortex
Source: Sci Rep. 2019 Mar 15;9:4619. doi: 10.1038/s41598-019-40549-y (PMC6420657; doi:10.1038/s41598-019-40549-y)

Fear conditioning and extinction induce opposing changes in dendritic spine remodeling and somatic activity of layer 5 pyramidal neurons in the mouse motor cortex

Zhiwei Xu[1, 2], Avital Adler[2], Hong Li[1], Luis M. Pérez-Cuesta[2], Baoling Lai[1,2], Wei Li[1] & Wen-Biao Gan[2*]

[1]State Key Laboratory of Chemical Oncogenomics, Key Laboratory of Chemical Genomics, Peking University Shenzhen Graduate School, Shenzhen, 518055, China. [2]Skirball Institute, Department of Neuroscience and Physiology, Department of Anesthesiology, New York University School of Medicine, New York, NY 10016, USA.

**Corresponding author. E-mail: gan@saturn.med.nyu.edu*

Supplementary Figure Legends

Figure S1. Inactivation of the primary motor cortex impairs conditioned freezing responses.
(A) Schematic of experimental design. Muscimol (0.2 μl, 1 μg/μl) or vehicle was infused bilaterally into the primary motor cortex prior to fear conditioning by pairing CS (1 kHz auditory tone) and US on day 0. The same mice were subjected to recall test on day 2.
(B) Mice with bilateral infusion of muscimol into the primary motor cortex before fear conditioning showed a significant increase in freezing during fear conditioning on day 0 (Two-Way ANOVA: drug: $F_{1,98} = 79.78$, $P < 0.0001$; trial: $F_{6,98} = 20.07$, $P < 0.0001$; interaction: $F_{6,98} = 4.576$, $P = 0.0004$) (n = 7 and 9 mice for muscimol and vehicle groups respectively).
(C) Bilateral infusion of muscimol into the primary motor cortex before fear conditioning significantly reduced freezing responses during the recall test on day 2 (n = 7 and 8 mice for muscimol and vehicle groups respectively). The freezing response of each trial during the recall test was used in the comparison.
Data are presented as mean ± S.E.M.

Figure S2. Infusion of muscimol into the primary motor cortex after fear conditioning does not significantly change conditioned freezing responses during the recall test.
(A) Schematic of experimental design. Muscimol (1 μl, 1 μg/μl) or vehicle was infused bilaterally into the primary motor cortex immediately after fear conditioning by pairing CS (1 kHz auditory tone) and US on day 0. The same mice were subjected to recall test on day 2.
(B) The freezing responses during the recall test were comparable between muscimol and vehicle groups (n = 7 and 8 mice for muscimol and vehicle groups respectively).
Data are presented as mean ± S.E.M.

Figure S3. Infusion of muscimol into the primary motor cortex before recall test does not prevent mice from showing freezing responses during the recall test.
(A) Schematic of experimental design. Muscimol (1 μl, 1 μg/μl) or vehicle was infused bilaterally into the primary motor cortex prior to recall test on day 2. The same mice were then subjected to recall test.
(B) The freezing responses during the recall test in muscimol-infused mice were similar to that in vehicle-infused mice (n = 7 and 8 mice for muscimol and vehicle groups respectively).

Data are presented as mean ± S.E.M.

Figure S4. The rate of spine elimination after fear conditioning in muscimol-infused mice is lower than that in vehicle-infused mice.

(A) Schematic of experimental design. Muscimol (1 μl, 1 μg/μl) or vehicle was injected bilaterally into the primary motor cortex prior to fear conditioning. Imaging was performed before muscimol injection and 2 days after fear conditioning.

(B) Muscimol-injected mice showed significantly lower spine elimination after fear conditioning than vehicle-injected mice (n = 4 mice for muscimol and vehicle groups respectively).

Data are presented as mean ± S.E.M. * $P < 0.05$.

Supplementary Figure S1

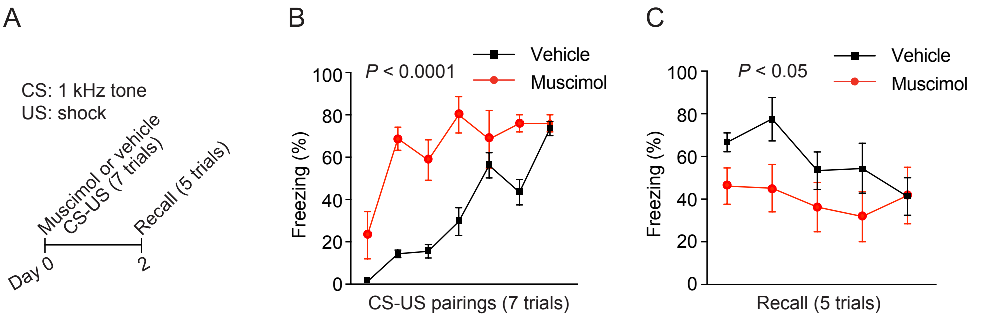

Supplementary Figure S2

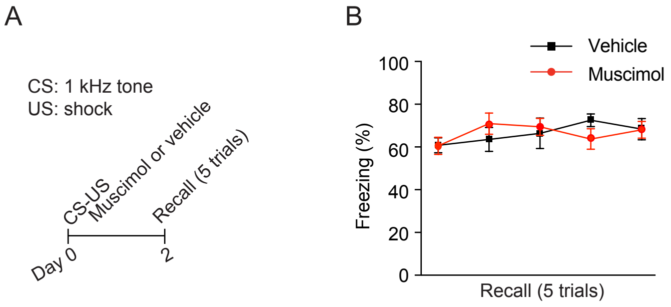

Supplementary Figure S3

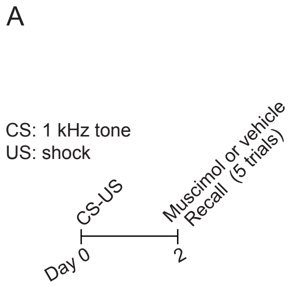

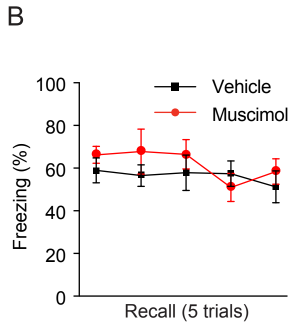

Supplementary Figure S4

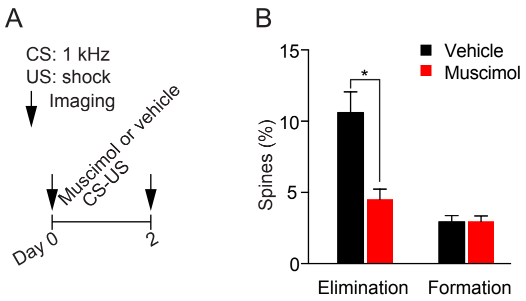

Supplement: Supplementary file 1 — Supplementary figures [file 41598_2019_40549_MOESM1_ESM.pdf]
